# Antimicrobial Resistance: Beyond the Breakpoint

**DOI:** 10.3201/eid1609.100788

**Published:** 2010-09

**Authors:** David J. Pombo

**Affiliations:** Author affiliation: Latter Day Saints Hospital, Salt Lake City, Utah, USA

**Keywords:** antimicrobial resistance, antibiotic, bacteria, viruses, fungi, book review

The Fifth Decennial International Conference on Healthcare-Associated Infections, held March 2010 in Atlanta, Georgia, USA, brought the issue of antimicrobial drug resistance to the forefront. Results of a survey that polled members of the Society for Healthcare Epidemiology of America regarding critical knowledge gaps and research priorities were presented. Respondents reported that 3 of the 5 most important issues they face are directly associated with antimicrobial drug resistance: multidrug–resistant gram-negative bacteria, antimicrobial stewardship, and methicillin-resistant *Staphylococcus aureus* (MRSA)

The publication of this collection of authoritative reviews of these issues is therefore timely. This concise volume draws on the knowledge of 23 authors, many of whom are either current or past staff members of the Centers for Disease Control and Prevention. In 10 chapters and 174 pages, these authors address contemporary issues in bacterial, fungal, parasitic, and viral (HIV) resistance, as well as some aspects of the effects of antimicrobial drug resistance on healthcare facilities. The main emphasis of the book is on the epidemiology and mechanisms and public health implications of resistant pathogens, not on details of treatment. Chapters on MRSA, extended-spectrum β-lactamase–producing gram-negative bacteria, and fluoroquinolone resistance cover the epidemiology and mechanisms of resistance, laboratory detection, and treatment considerations. A historical review of the contribution of bacterial pneumonia to the death rate of previous influenza pandemics clearly discusses the likelihood and consequences of resistance in strains of *Streptococcus pneumoniae* and *S. aureus*. Questions raised here about how these antimicrobial drug–resistant bacteria affect a modern pandemic, such as the impact of antiviral drugs and diagnostic testing on antibiotic use, can now be investigated in light of the subsequent pandemic (H1N1) 2009.

Three chapters address resistance in healthcare settings and the promotion of appropriate antimicrobial drug use. The first describes interventions to reduce the inappropriate use of antimicrobial agents for respiratory conditions and quality initiatives that can improve prescribing. The chapter that reviewed effective strategies for controlling resistant pathogens in hospitals is the only chapter that was not well referenced on some of the more controversial issues, such as silver coating of devices. However, the chapter on estimating costs attributable to infections caused by antimicrobial drug–resistant bacteria is comprehensive.

Three final chapters expand the scope of this volume into issues of parasitic, fungal, and viral resistance. Resistance in helminths is presented in the context of mass treatment during eradication programs and the need for enhanced surveillance programs. The chapter on antifungal resistance reviews available drugs and laboratory detection of resistance. The final chapter on HIV drug resistance in the developing world reviews initial concerns and current encouraging data on antiretroviral drug–resistance in sub-Saharan Africa and southern Asia. Current public health strategies for detecting and controlling drug–resistant HIV are given, along with a clear account of the biological and pharmacologic factors that affect HIV resistance and a review of areas needing continued attention and resources.

The strength of this book is the wide scope of its coverage of antimicrobial drug resistance. Most chapters are well written in a succinct style and format easily accessible to the general reader. The text has supportive references from primary sources, and contains a good index. This book is a valuable resource for those beginning their careers or who are looking for a research focus, or for anyone already an expert in an aspect of antimicrobial resistance who is seeking a broader perspective.

